# Clinical characteristics of patients with high extracellular volume fraction evaluated by cardiac computed tomography for coronary artery evaluation

**DOI:** 10.1093/ehjopen/oeae036

**Published:** 2024-04-27

**Authors:** Tetsuya Oguni, Seiji Takashio, Naoto Kuyama, Kyoko Hirakawa, Shinsuke Hanatani, Fumi Oike, Hiroki Usuku, Yasushi Matsuzawa, Masafumi Kidoh, Seitaro Oda, Eiichiro Yamamoto, Mitsuharu Ueda, Toshinori Hirai, Kenichi Tsujita

**Affiliations:** Department of Cardiovascular Medicine, Graduate School of Medical Sciences, Kumamoto University, 1-1-1 Honjo, 860-8556 Kumamoto, Japan; Department of Cardiovascular Medicine, Graduate School of Medical Sciences, Kumamoto University, 1-1-1 Honjo, 860-8556 Kumamoto, Japan; Department of Cardiovascular Medicine, Graduate School of Medical Sciences, Kumamoto University, 1-1-1 Honjo, 860-8556 Kumamoto, Japan; Department of Cardiovascular Medicine, Graduate School of Medical Sciences, Kumamoto University, 1-1-1 Honjo, 860-8556 Kumamoto, Japan; Department of Cardiovascular Medicine, Graduate School of Medical Sciences, Kumamoto University, 1-1-1 Honjo, 860-8556 Kumamoto, Japan; Department of Cardiovascular Medicine, Graduate School of Medical Sciences, Kumamoto University, 1-1-1 Honjo, 860-8556 Kumamoto, Japan; Department of Cardiovascular Medicine, Graduate School of Medical Sciences, Kumamoto University, 1-1-1 Honjo, 860-8556 Kumamoto, Japan; Department of Cardiovascular Medicine, Graduate School of Medical Sciences, Kumamoto University, 1-1-1 Honjo, 860-8556 Kumamoto, Japan; Department of Diagnostic Radiology, Graduate School of Medical Sciences, Kumamoto University, Kumamoto, Japan; Department of Diagnostic Radiology, Graduate School of Medical Sciences, Kumamoto University, Kumamoto, Japan; Department of Cardiovascular Medicine, Graduate School of Medical Sciences, Kumamoto University, 1-1-1 Honjo, 860-8556 Kumamoto, Japan; Department of Neurology, Graduate School of Medical Sciences, Kumamoto University, Kumamoto, Japan; Department of Diagnostic Radiology, Graduate School of Medical Sciences, Kumamoto University, Kumamoto, Japan; Department of Cardiovascular Medicine, Graduate School of Medical Sciences, Kumamoto University, 1-1-1 Honjo, 860-8556 Kumamoto, Japan; Center of Metabolic Regulation of Healthy Aging, Faculty of Life Sciences, Kumamoto University, Kumamoto, Japan

**Keywords:** Cardiac computed tomography, Extracellular volume fraction, Cardiac amyloidosis

## Abstract

**Aims:**

This study aims to evaluate the distribution of extracellular volume fraction detected via computed tomography, clinical characteristics of high extracellular volume fraction detected via computed tomography, and the rate of incidental detection of cardiac amyloidosis in patients undergoing cardiac computed tomography for coronary artery evaluation.

**Methods and results:**

This study included 874 consecutive patients (mean age, 74.4 ± 7.1 years; men, 65%), comprising men aged ≥60 years and women aged ≥70 years, who had undergone cardiac computed tomography between January 2020 and September 2022. The mean extracellular volume fraction detected via computed tomography was 29.7 ± 5.2%, and 108 patients (12.4%) had an extracellular volume fraction detected via computed tomography of ≥35%. Older age (75.9 ± 8.2 years vs. 74.2 ± 6.9 years; *P* = 0.042), male sex (75.9% vs. 63.0%; *P* = 0.007), impaired left ventricular ejection fraction, increased high-sensitivity cardiac troponin T and B-type natriuretic peptide levels, and increased left ventricular thickness showed significant associations with an extracellular volume fraction detected via computed tomography of ≥35%. Cardiac amyloidosis was diagnosed incidentally in 15 patients based on an increase in extracellular volume fraction detected via computed tomography. The prevalence of cardiac amyloidosis was 1.7% (15/874) and 14.3% (15/105) in the entire study population and in patients with an extracellular volume fraction detected via computed tomography of ≥35%, respectively. An increase in the extracellular volume fraction detected via computed tomography was suggestive of cardiac amyloidosis.

**Conclusion:**

Elevated extracellular volume fraction detected via computed tomography, associated with elevated cardiac biomarker levels and myocardial structural changes, may lead to the incidental diagnosis of cardiac amyloidosis.

## Graphical Abstract

**Graphical Abstract:**
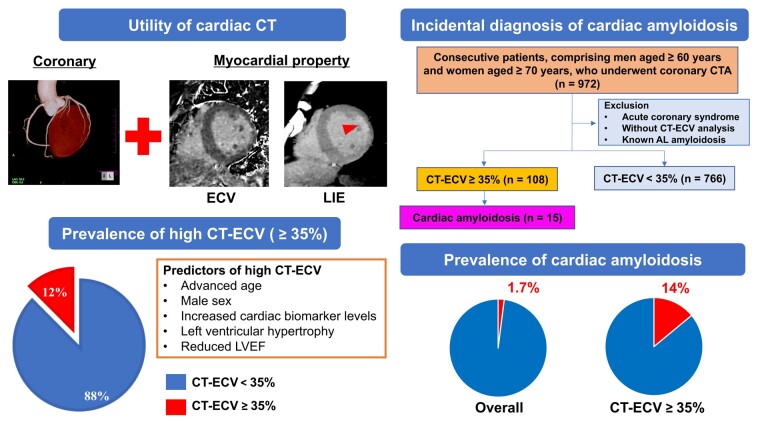
Cardiac computed tomography is useful for evaluating myocardial properties using extracellular volume by computed tomography. High extracellular volume by computed tomography is associated with increased levels of cardiac biomarkers and structural changes in the myocardium. Cardiac amyloidosis was incidentally diagnosed in approximately 1.7% of patients who underwent cardiac computed tomography for coronary artery evaluation. CCT, cardiac computed tomography; CT-ECV, extracellular volume by computed tomography; LIE, late iodine enhancement; CTA, computed tomography angiography.

## Introduction

Diagnostic imaging in cardiomyopathy care has attracted considerable attention in recent years. Cardiac magnetic resonance (CMR) enables the evaluation of cardiac function, morphology by cine, and myocardial properties via late contrast enhancement, T1 mapping, and determination of the myocardial extracellular volume (ECV) fraction. Extracellular volume can be used to quantitatively evaluate the extracellular matrix, and it aids in the diagnosis and prognostic evaluation of cardiomyopathy and its association with the exacerbation of heart failure.^1,2^ However, CMR is associated with a long imaging time. Moreover, its use is limited in patients with cardiac implantable electronic devices and those undergoing dialysis. In clinical practice, obtaining cardiac images that lead to a simple diagnosis in a short time without use limitations is necessary.

Coronary computed tomography angiography (CTA) is used for the evaluation of anatomical coronary artery lesions. Patients with cardiac implantable electronic devices and patients undergoing dialysis can undergo coronary CTA; thus, the number of patients undergoing coronary CTA is increasing. Late contrast enhancement and ECV by computed tomography (CT-ECV) have been used for imaging the delayed phase after coronary CTA in recent years. Extracellular volume by computed tomography shows excellent correlation with MRI-ECV.^3^ Extracellular volume by computed tomography is evaluated by adding a delayed phase to cardiac computed tomography (CCT) performed for pre-transcatheter aortic valve implantation (TAVI) planning and pre-atrial fibrillation ablation planning, and the usefulness of CT-ECV in the detailed examination and prognosis prediction after therapeutic intervention in patients with cardiomyopathy has been reported.^4–6^ Thus, CT-ECV has attracted attention for myocardial characterization. Cardiac amyloidosis is a cardiomyopathy characterized by a marked elevation in ECV. Cardiac amyloidosis should be actively suspected in patients with ECV >40%.^7^ Approximately 23% of patients are diagnosed with wild-type transthyretin cardiac amyloidosis (ATTR-CA) based on incidental findings detected during imaging examinations, such as echocardiography and bone scintigraphy.^8^ Performing CT-ECV simultaneously with coronary CTA and examining patients with high CT-ECV values may lead to an early diagnosis of cardiac amyloidosis; however, this hypothesis has not been fully evaluated.

This study aimed to examine the clinical characteristics of patients with high CT-ECV and the prevalence of incidental diagnosis of cardiac amyloidosis in patients who underwent coronary CTA for the evaluation of the coronary arteries.

## Methods

This single-centre, retrospective study conformed to the principles outlined in the Declaration of Helsinki and was approved by the Institutional Review Board and Ethics Committee of Kumamoto University (approval no. 2384). The requirement for obtaining informed consent was waived owing to the low-risk nature of the study and the inability to obtain consent directly from all participants. The study protocol was posted on the hospital website (https://kumadai-radiology.jp/medical-research/) and at the Kumamoto University Hospital to provide patients with the opportunity to withdraw participation.

### Study population

The clinical records of patients with suspected coronary artery disease who underwent coronary CTA at our institution between January 2020 and September 2022 were retrospectively reviewed. Among the patients who underwent coronary CTA during this period, men and women aged ≥60 years and ≥70 years, respectively, were included in this study. Patients with suspected coronary artery disease and those with confirmed coronary artery disease underwent coronary CTA for clinical reasons based on the guidelines of the European Society of Cardiology. The exclusion criteria were as follows: (i) acute coronary syndrome, (ii) CT-ECV not performed, and (iii) cardiac amyloidosis diagnosed before undergoing coronary CTA. The cut-off value for abnormal ECV has not been determined; thus, the cut-off value was set as 35% at our institution, based on our clinical experience.

### Cardiac computed tomography imaging protocol

**Figure 1:**
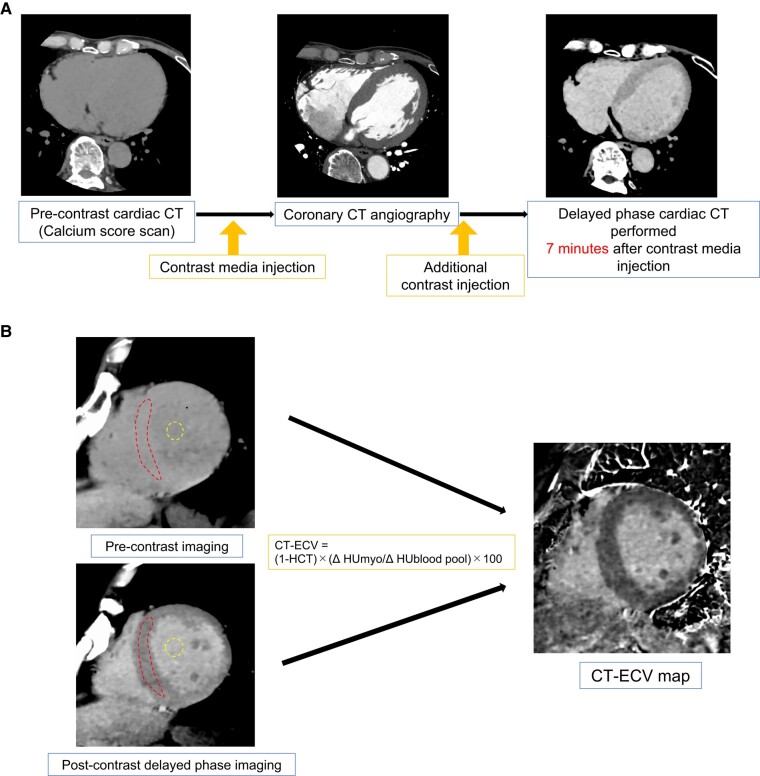
Cardiac computed tomography angiography protocol and extracellular volume analysis. Timeline illustrating the anatomical coronary artery and extracellular volume quantification computed tomography protocol. The delayed-phase scan was acquired 7 min after the intravenous injection of iodinated contrast medium (*A*). The diagram shows the extracellular volume measurement method. The extracellular volume by computed tomography was calculated as extracellular volume by computed tomography = (1 − haematocrit) × (Δ myo/Δ blood pool) × 100. Regions of interest are drawn manually on the septal segment of the midventricular short-axis multi-planar reconstruction images of the extracellular volume by computed tomography map (*B*). CT, computed tomography; ECV, extracellular volume; HCT, haematocrit; Δ myo, myocardial Hounsfield units (post—pre-contrast); Δ blood pool, left ventricular Hounsfield units (post–pre-contrast).

Delayed-phase CCT (equilibrium-phase image acquisition) for CT-ECV measurement is performed as part of the routine clinical coronary CTA protocol at our institution. A 320 × 0.5 mm detector-row CT unit (Aquilion One Genesis edition; Canon Medical Systems) was used for CT examinations. The CCT protocol at our institution involves the acquisition of a topogram, pre-contrast CCT (calcium score scan), coronary CTA, and delayed-phase CCT. Pre-contrast CCT was performed at 120 kVp [0.275 ms/rot, tube current = 750 mA, volume CT scan dose index (CTDI_vol_) = 10.6 mGy, and dose-length product (DLP) = 169 mGy·cm in representative cases] in mid-diastole (75% of the R-R intervals) with low heart rates or during end-systole (30–40% of the R-R intervals) with high heart rates (>70 beats per minute). Coronary CTA was performed subsequently during the optimal cardiac phase, using the same cardiac phase as that in the pre-contrast CCT. A contrast material dose of 450 mg I/kg was used for coronary CTA. An additional amount of contrast material was infused immediately after coronary CTA and was administered at a rate of 1.5 mL/s. Approximately 550 mg I/kg of body weight iodine (Iopamiron 370; Bayer Healthcare) was used. Lastly, delayed-phase cardiac CT was performed at 120 kVp (0.275 ms/rot) in the same cardiac phase as the pre-contrast CCT 7 min after the first injection of the contrast medium. The maximum tube current [750 mA (fixed), CTDI_vol_ = 11.6 ± 0 mGy, DLP = 186 ± 0 mGy·cm] was used to reduce the image noise and artefacts in delayed-phase cardiac CT (*Figure 1A*).

### Extracellular volume by computed tomography analysis

Myocardial CT-ECV quantification was performed on pre-contrast and post-contrast delayed-phase CT images using the subtraction method.^9^ To create the CT-ECV maps, pre- and post-contrast images were analysed using a dedicated application installed on a post-processing workstation (Ziostation2; Ziosoft, Tokyo, Japan). The myocardial CT-ECV was calculated using the following formula:


CT-ECV=(1–haematocrit)×(ΔHUmyo/ΔHUbloodpool)×100,

where Δ myo is the change in the myocardial Hounsfield units (post-contrast–pre-contrast) and Δ blood pool is the change in the left ventricular Hounsfield units (post-contrast–pre-contrast). Regions of interest (ROIs) were manually drawn on the septal segment of the midventricular short-axis multi-planar reconstruction images of the CT-ECV map. Each myocardial ROI was drawn to be as large as possible to include the septal segment from the epicardium to the endocardium. Only the myocardial pixels that were not contaminated by the in-plane partial volume effect of blood, epicardial fat, or pericardial fluid were included in the manually drawn regions (*Figure 1B*).

### Biomarker analysis

The serum high-sensitivity cardiac troponin T (hs-cTnT) levels (normal cut-off value: 0.014 pg/mL) were measured at the time of diagnosis using the Elecsys 2010 Troponin T hs kit (Roche Diagnostics, Indianapolis, IN, USA). The plasma B-type natriuretic peptide (BNP) levels (normal cut-off value: 18.4 pg/mL) were measured using the MI02 Shionogi BNP kit (Abbott Japan, Matsudo, Japan). The hs-cTnT and BNP data were obtained within 60 days of CT examination under stable conditions. The glomerular filtration rate (GFR) was calculated using the Modification of Diet in Renal Disease Study equation, level modified for Japanese individuals.

### Echocardiographic analysis

Echocardiography was performed using commercially available ultrasound equipment, including Vivid E95 or 7 (GE Vingmed, Horten, Norway), Aplio 500 (Canon Medical Systems Corp., Otawara, Tochigi, Japan), and Epiq 7G (Philips, Bothell, WA, USA). The most recent echocardiographic data within 60 days of the CT examination were obtained. The size of the cardiac chamber and the thickness of the walls were determined using the transthoracic view. The left ventricular (LV) ejection fraction (LVEF) was calculated using the modified Simpson’s method.

### Diagnosis of cardiac amyloidosis

Amyloid deposition was detected via Congo red staining and the detection of apple-green birefringence using cross-polarized light microscopy. Detailed diagnostic examinations for cardiac amyloidosis, including pathological and imaging evaluations, were performed at the discretion of the attending physicians. Transthyretin cardiac amyloidosis was diagnosed based on (i) the presence of transthyretin (TTR) amyloid deposition in the myocardium or extracardiac tissue, such as subcutaneous tissue or gastrointestinal tract, with a positive finding of ^99m^Tc-labelled pyrophosphate (PYP) scintigraphy. Light-chain (AL) amyloidosis was excluded based on the results of serum and urine protein electrophoresis by immunofixation electrophoresis (definite ATTR-CA). (ii) Positive ^99m^Tc-labeled PYP scintigraphy findings without the confirmation of pathological TTR deposition and exclusion of AL amyloidosis (probable ATTR-CA).^10,11^ Light-chain cardiac amyloidosis was diagnosed based on the detection of AL amyloid deposition in the myocardium or extracardiac tissue, with typical findings of cardiac amyloidosis, such as a thickened LV wall on echocardiography or CMR. Suspect cardiac amyloidosis was diagnosed based on the echocardiographic findings of non-dilated hypertrophic LV and LV diastolic failure (non-dilated hypertrophic LV: LVEF ≥ 50%; LV interventricular septal thickness of ≥12 mm in women and ≥13 mm in men, and LV end-diastolic volume index of <85 mL/m^2^) without pathological, serological, and imaging evaluation for cardiac amyloidosis.^12^ The incidental diagnosis of cardiac amyloidosis was defined as a diagnosis triggered by an increase in CT-ECV.

### Statistical analysis

Statistical analyses were performed using IBM SPSS Statistics version 29 (IBM Corporation, Armonk, New York, USA). Normally distributed parameters are expressed as mean ± standard deviation, whereas non-normally distributed parameters are expressed as median with interquartile range (IQR). Categorical values are presented as numbers (percentages). Student’s *t*-test or the Mann–Whitney *U* test was used to compare continuous variables, and the chi-squared test for categorical data was used as appropriate. The correlation between CT-ECV and the clinical parameters was assessed using Pearson’s analysis. The BNP and hs-cTnT levels were logarithmically transformed before calculating Pearson’s correlation coefficient, as they were not normally distributed. Univariate and multivariate analyses were performed using binary logistic regression, using the presence of CT-ECV ≥ 35% as the dependent variable. As the factors contributing to high CT-ECV have not been established, variables for multivariate analysis were selected based on the statistical significance in univariate analysis and clinical relevance, while avoiding multi-collinearity. Multi-collinearity was determined using a variance inflation factor of >10. The goodness of fit test was determined using the Hosmer–Lemeshow model. A two-tailed *P*-value of <0.05 was considered statistically significant.

## Results

### Clinical characteristics of the patients

**Figure 2:**
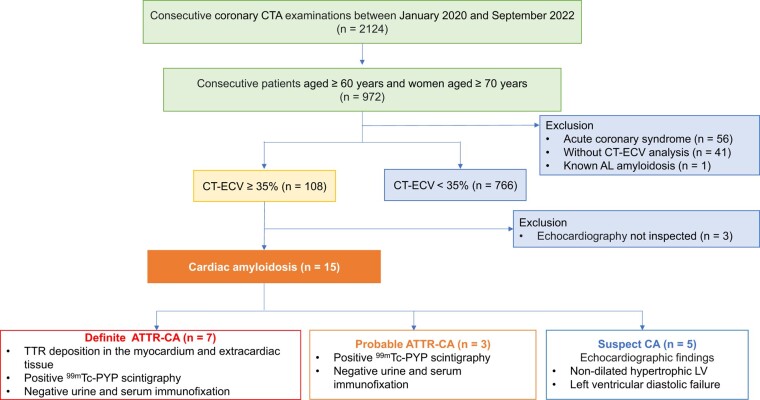
Study flow chart detailing the inclusion and exclusion criteria for study patients. AL, amyloid light chain; CT-ECV, extracellular volume by computed tomography; CTA, computed tomography angiography; ATTR-CA, transthyretin cardiac amyloidosis; CA, cardiac amyloidosis; ^99m^Tc-PYP, ^99m^Tc-labeled pyrophosphate; LV, left ventricle.

**Figure 3:**
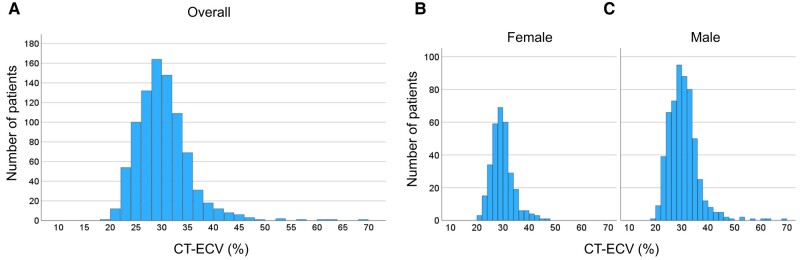
Histogram of extracellular volume by computed tomography. The histogram shows the extracellular volume by computed tomography distribution in 875 patients who underwent cardiac computed tomography: (*A*) overall, (*B*) female, and (*C*) male. CT-ECV, extracellular volume by computed tomography.

*Figure 2* presents the patient selection process. Among the 2124 consecutive patients who underwent coronary CTA between January 2020 and September 2022, 972 patients, comprising men aged ≥60 years and women aged ≥70 years, were included in this study. Ninety-eight patients were excluded for the following reasons: acute coronary syndrome (*n* = 56), CT-ECV analysis not performed (*n* = 41), or known diagnosis of AL cardiac amyloidosis (*n* = 1). Thus, the study cohort comprised 874 patients (mean age, 74.4 ± 7.1 years; men, 65%). The patients’ clinical characteristics are presented in *Table 1*. The mean CT-ECV was 29.8 ± 5.2%, and the distribution is presented in *Figure 3*.

**Table 1:** Baseline characteristics of the patients

	All	CT-ECV < 35%	CT-ECV ≥ 35% (*n* = 108)	*P*-value
	(*n* = 874)	(*n* = 766)		
Demographics				
Male, *n* (%)	565 (65)	483 (63)	82 (76)	0.007
Age, years	74.4 ± 7.1	74.2 ± 6.9	75.9 ± 8.2	0.042
Medical history				
Hypertension, *n* (%)	598 (68)	517 (67)	81 (75)	0.104
Diabetes mellitus, *n* (%)	298 (34)	245 (32)	53 (49)	<0.001
Dyslipidaemia, *n* (%)	472 (54)	412 (54)	60 (56)	0.805
Atrial fibrillation, *n* (%)	148 (17)	126 (16)	22 (20)	0.331
Previous revascularization				
PCI and/or CABG	208 (24)	171 (22)	37 (34)	0.011
Laboratory examination parameters				
Haematocrit, %	39.3 ± 5.9	39.8 ± 5.7	36.4 ± 5.9	<0.001
hs-cTnT, ng/mL	0.013 (0.009–0.023)	0.012 (0.008–0.020)	0.026 (0.017–0.061)	<0.001
BNP, pg/mL	45.6 (19.8–128.7)	42.5 (17.5–99.3)	171.1 (47.9–331.7)	<0.001
Sodium, mEq/L	139.9 ± 2.7	140 ± 2.6	139.2 ± 3.1	0.011
Potassium, mEq/L	4.4 ± 3.6	4.4 ± 3.8	4.3 ± 0.6	0.678
eGFR, mL/min/1.73m^2^	58.0 ± 18.8	59.3 ± 17.4	48.6 ± 24.2	<0.001
Echocardiogram parameters				
LVEF, %	58.1 ± 9.8	58.9 ± 9.2	52.3 ± 11.4	<0.001
LVDd, mm	44.8 ± 6.9	44.4 ± 6.4	47.3 ± 9.2	0.002
LVDs, mm	30.3 ± 7.9	29.6 ± 7.2	34.7 ± 10.3	<0.001
Intraventricular septal thickness, mm	10.5 ± 1.9	10.4 ± 1.8	11.2 ± 2.4	0.003
LV posterior wall thickness, mm	10.2 ± 1.7	10.0 ± 1.5	10.8 ± 2.4	0.004
E/A ratio	1.1 ± 3.7	1.0 ± 3.7	1.3 ± 2.9	0.442
*E*/*e*′ ratio	13.5 ± 6.8	13.0 ± 6.1	16.7 ± 9.8	<0.001
CT measurements				
Extracellular volume, %	29.8 ± 5.2	28.3 ± 3.3	39.5 ± 5.9	<0.001
Coronary artery stenosis, *n* (%)	390 (45)	337 (44)	53 (49)	0.410

Values are presented as the number of patients (%), mean ± standard deviation (SD), and median (interquartile range).

BNP, B-type natriuretic peptide; CABG, coronary artery bypass graft; CT, computed tomography; CT-ECV, extracellular volume by computed tomography; eGFR, estimated glomerular filtration rate; hs-cTnT, high-sensitivity cardiac troponin T; LV, left ventricle; LVDd, left ventricular diastolic diameter; LVDs, left ventricular systolic diameter; LVEF, left ventricular ejection fraction; PCI, percutaneous coronary intervention.

A total of 108 patients (12.4%) had CT-ECV ≥ 35%. Compared with patients with CT-ECV < 35%, those with CT-ECV ≥ 35% were significantly older (75.9 ± 8.2 years vs. 74.2 ± 6.9 years; *P* = 0.042) and males (75.9% vs. 63.0%; *P* = 0.007). Moreover, the cardiac biomarkers levels were significantly elevated in these patients [BNP: 171.1 (IQR: 47.9–331.7) pg/mL vs. 42.5 (IQR: 17.5–99.3) pg/mL; *P* < 0.001; hs-cTnT: 0.026 (0.017–0.061) ng/mL vs. 0.012 (IQR: 0.008–0.020) ng/mL; *P* < 0.001]. The intraventricular septal thickness (IVST) was significantly higher (11.2 ± 2.4 mm vs. 10.4 ± 1.8 mm; *P* = 0.003), whereas LVEF was significantly lower (52.3 ± 11.4% vs. 58.9 ± 9.2%; *P* < 0.001) in patients with CT-ECV ≥ 35%.

### Patterns of late contrast enhancement

**Figure 4:**
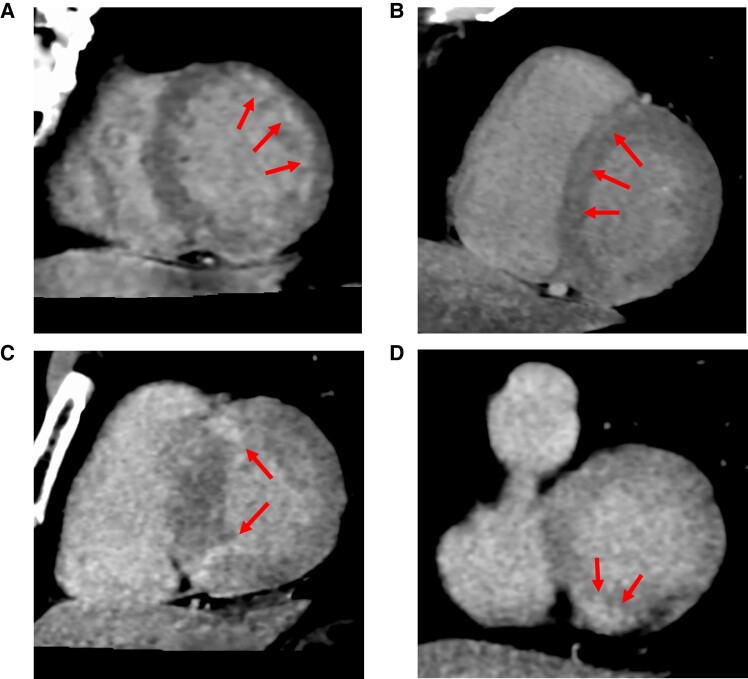
Late contrast enhancement patterns. Examples of various late contrast enhancement patterns: (*A*) subendocardial, (*B*) septal mid-wall, (*C*) inferior and superior left ventricular/right ventricular junction, and (*D*) epicardial pattern.

The late contrast enhancement patterns observed in the 324 were classified as follows: sub-endocardial or transmural (*n* = 195, 60.2%), mid-wall (*n* = 60, 18.5%), inferior and superior LV/RV junctions (*n* = 36, 11.1%), and other (focal or epicardial; *n* = 33, 10.2%) patterns (*Figure 4*).

### Predictors of extracellular volume by computed tomography ≥ 35%

Univariate analysis performed to identify the predictors of CT-ECV ≥ 35% identified older age; male sex; history of diabetes mellitus; previous revascularization; estimated GFR; hs-cTnT, BNP, and sodium levels; LV diastolic diameter; LV systolic diameter; IVST; LV posterior wall thickness; LVEF; and *E*/*e*′ as predictors of CT-ECV ≥ 35%. Multivariate analysis revealed that age, male sex, and the hs-cTnT and BNP levels were significantly associated with CT-ECV ≥ 35% (*Table 2*). Coronary artery stenosis was not associated with CT-ECV ≥ 35%.

**Table 2:** Univariate and multivariate binary logistic regression analyses based on extracellular volume by computed tomography of ≥35%

	Univariate analysis			Multivariate analysis		
	Exp (B)	95% CI for Exp (B)	*P*-value	Exp (B)	95% CI for Exp (B)	*P*-value
Male	1.85	1.16–2.94	0.010	2.13	1.14–4.01	0.018
Age (per year increase)	1.03	1.01–1.06	0.020	1.05	1.01–1.09	0.009
Hypertension	1.45	0.91–2.29	0.118			
Diabetes mellitus	2.05	1.36–3.01	<0.001	1.49	0.83–2.65	0.181
Dyslipidaemia	1.07	0.72–1.61	0.730			
Atrial fibrillation	1.30	0.78–2.16	0.310			
PCI and/or CABG	1.81	1.18–2.79	0.007	0.72	0.38–1.35	0.305
Ln (hs-cTnT), per 1 ng/mL increment	2.10	1.71–2.59	<0.001	1.53	1.11–2.10	0.009
Ln (BNP), per 1 pg/mL increment	1.90	1.61–2.23	<0.001	1.40	1.05–1.85	0.022
Haematocrit	0.90	0.87–0.94	<0.001			
eGFR, per 1 mL/min/1.73 m^2^ increment	0.97	0.96–0.98	<0.001	1.01	0.99–1.02	0.536
Sodium, per 1 mEq/L increment	0.90	0.84–0.96	0.003	0.93	0.85–1.02	0.132
Potassium, per 1 mEq/L increment	0.94	0.62–1.41	0.751			
LV ejection fraction, per 10% decrement	0.95	0.93–0.96	<0.001	0.98	0.95–1.01	0.245
LVDd, per 1 mm increment	1.06	1.03–1.09	<0.001	1.00	0.95–1.05	0.989
LVDs, per 1 mm increment	1.07	1.05–1.10	<0.001			
Intraventricular septal thickness, per 1 mm increment	1.20	1.08–1.32	<0.001	1.09	0.95–1.24	0.211
LV posterior wall thickness, per 1 mm increment	1.24	1.11–1.39	<0.001			
*E*/*A* ratio, per increment	1.02	0.97–1.06	0.472			
*E*/*e*′ ratio, per increment	1.06	1.03–1.09	<0.001	1.02	0.99–1.05	0.271
Coronary artery stenosis	1.23	0.82–1.84	0.321			

BNP, B-type natriuretic peptide; CABG, coronary artery bypass graft; CI, confidence interval; CT, computed tomography; CT-ECV, extracellular volume by computed tomography; eGFR, estimated glomerular filtration rate; Exp (B), exponentiation of the B coefficient; hs-cTnT, high-sensitivity cardiac troponin T; LV, left ventricle; LVDd, left ventricular diastolic diameter; LVDs, left ventricular systolic diameter; LVEF, left ventricular ejection fraction; PCI, percutaneous coronary intervention.

Extracellular volume by computed tomography was found to be correlated with the cardiac biomarkers BNP (*r* = 0.360, *P* < 0.001) and hs-cTnT (*r* = 0.349, *P* < 0.001) levels, as well as the echocardiographic parameters LVEF (*r* = −0.261, *P* < 0.001) and IVST (*r* = 0.173, *P* < 0.001).

### Incidental diagnosis of cardiac amyloidosis

Among the 874 patients included in this study, seven, three, and five patients were diagnosed with ‘definite ATTR-CA’, ‘probable ATTR-CA’, and ‘suspect cardiac amyloidosis’, respectively. None of the patients had AL cardiac amyloidosis. The attending physicians did not suspect cardiac amyloidosis prior to coronary CTA in any of the patients. Among the patients with CT-ECV ≥ 35%, the diagnosis of cardiac amyloidosis was ruled out in 11.4% (12/105) and 3.8% (4/105) patients via CMR and ^99m^Tc-PYP scintigraphy, respectively. The prevalence of cardiac amyloidosis was estimated to be at least 1.7% (15/874) in the entire study population and 14.3% (15/105) among the patients with CT-ECV ≥ 35%. The prevalence of cardiac amyloidosis was 1.1% (10/874) in the entire study population and 9.5% (10/105) among the patients with CT-ECV ≥ 35% when ‘suspect cardiac amyloidosis’ was excluded. No patient was diagnosed with cardiac amyloidosis with CT-ECV < 35%.

**Figure 5:**
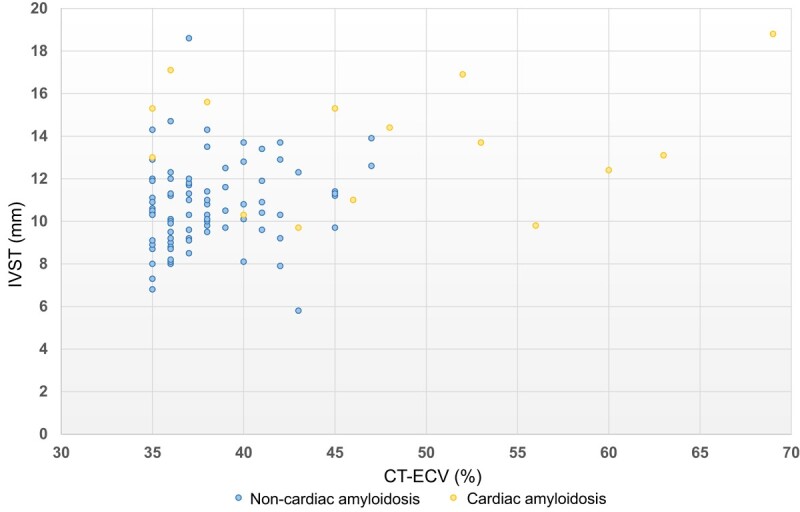
Distribution of extracellular volume by computed tomography and interventricular septal thickness in patients with extracellular volume by computed tomography ≥ 35%. The scatterplot shows the distribution of extracellular volume by computed tomography and interventricular septal thickness in patients with extracellular volume by computed tomography of ≥35%. The yellow circle represents the patients diagnosed with cardiac amyloidosis. CT-ECV, extracellular volume by computed tomography; IVST, interventricular septal thickness.

*Table 3* presents the comparison of the characteristics between the non-cardiac amyloidosis and cardiac amyloidosis groups with CT-ECV ≥ 35%. The mean CT-ECV in the cardiac amyloidosis group was significantly higher than that in the non-cardiac amyloidosis group (47.9 ± 10.7% vs. 38.1 ± 3.1%; *P* = 0.003). Compared with the non-cardiac amyloidosis group, the cardiac amyloidosis group had thicker LV walls (*P* < 0.001). The scatterplot in *Figure 5* presents the distribution of CT-ECV and IVST among patients with CT-ECV ≥ 35%. Thirty-eight patients had LV septal thickening (IVST ≥ 12 mm), and 29% (11/38) of these patients had cardiac amyloidosis.

**Table 3:** Baseline characteristics of the patients with extracellular volume by computed tomography of ≥35% in the non-cardiac amyloidosis and cardiac amyloidosis groups

	Non-cardiac amyloidosis (*n* = 90)	Cardiac amyloidosis (*n* = 15)	*P*-value
Demographics			
Male, *n* (%)	70 (78)	12 (80)	0.847
Age, years	75.3 ± 8.5	78.1 ± 5.7	0.117
Medical history			
Hypertension, *n* (%)	67 (74)	12 (80)	0.644
Diabetes mellitus, *n* (%)	46 (51)	5 (33)	0.202
Dyslipidaemia, *n* (%)	49 (54)	9 (60)	0.689
Atrial fibrillation, *n* (%)	17 (19)	3 (20)	0.919
Previous revascularization			
PCI and/or CABG	32 (36)	4 (27)	0.502
Laboratory examination parameters			
Haematocrit, %	35.8 ± 5.8	40.1 ± 5.4	0.008
hs-cTnT, ng/mL	0.026 (0.016–0.062)	0.048 (0.019–0.075)	0.291
BNP, pg/mL	177.7 (47.8–343.5)	133.9 (66.8–266.1)	0.575
eGFR, mL/min/1.73 m^2^	47.6 ± 25.5	53.8 ± 17.2	0.248
Echocardiogram parameters			
LVEF, %	51.7 ± 11.7	55.6 ± 9.3	0.242
LVDd, mm	48.6 ± 9.2	40.0 ± 5.6	<0.001
LVDs, mm	35.7 ± 10.8	29.4 ± 5.7	0.002
Intraventricular septal thickness, mm	10.7 ± 2.0	13.8 ± 2.8	<0.001
LV posterior wall thickness, mm	10.3 ± 1.8	13.8 ± 3.2	<0.001
*E*/*A* ratio	1.4 ± 3.2	1.3 ± 0.9	0.960
*E*/*e*′ ratio	16.3 ± 10.3	19.0 ± 6.9	0.326
CT measurements			
Extracellular volume, %	38.1 ± 3.1	47.9 ± 10.7	0.003
Coronary artery stenosis, *n* (%)	43 (48)	8 (53)	0.690

Values are presented as the number of patients (%), mean ± standard deviation (SD), and median (interquartile range).

BNP, B-type natriuretic peptide; CABG, coronary artery bypass graft; CT, computed tomography; CT-ECV, extracellular volume by computed tomography; eGFR, estimated glomerular filtration rate; hs-cTnT, high-sensitivity cardiac troponin T; LV, left ventricle; LVDd, left ventricular diastolic diameter; LVDs, left ventricular systolic diameter; LVEF, left ventricular ejection fraction; PCI, percutaneous coronary intervention.

## Discussion

The present study investigated CT-ECV in men and women aged ≥60 and ≥70 years, respectively, who underwent coronary CTA for coronary artery evaluation. The major findings of this study included the following: (i) increased hs-cTnT and BNP levels, LV hypertrophy, and reduced LVEF were associated with elevated CT-ECV regardless of the presence of coronary artery stenosis; (ii) CCT can be used to evaluate coronary artery stenosis, myocardial properties, and injury using CT-ECV, and it serves as an opportunity for detailed examination of non-ischaemic cardiomyopathy; and (iii) increased CT-ECV is a particularly important predictor of the diagnosis of cardiac amyloidosis, and 1.7% of patients were incidentally diagnosed with cardiac amyloidosis based on an increase in CT-ECV.

### Clinical characteristics and practical uses of extracellular volume by computed tomography

Cardiac computed tomography is mainly used to evaluate coronary artery and structural heart disease, whereas CMR is mainly used to evaluate cardiac function and myocardial properties. However, CMR imaging has limited use in patients with cardiac implantable electronic devices. Extracellular volume by computed tomography offers key advantages over CMR as it can be easily added to routine coronary CTA through the simple addition of a post-contrast phase. Extracellular volume by computed tomography may be a more rapid, less expensive, and more widely available alternative to CMR; moreover, it may be advantageous in patients who cannot undergo CMR, such as patients with cardiac implantable electronic devices and patients undergoing haemodialysis.

Delayed-phase CCT for ECV imaging requires a long examination time, as well as additional radiation exposure and contrast material. The radiation exposure was set at sufficiently low levels to be considered justified. The risks and benefits of radiation exposure should be carefully considered, such that the exposure levels are ‘as low as reasonably achievable (ALARA principle)’ for all patients.^13^ An optimal contrast dose remains to be established for CT-ECV; therefore, additional amounts of the contrast material were administered based on individual patient risk factors, such as renal function. The amount of contrast material, which is adjusted based on estimated GFR (eGFR), is reduced by 20% in patients with eGFR ≤ 45 mL/min/1.73 m^2^, and, in principle, not used in patients with eGFR ≤ 30 mL/min/1.73 m^2^. However, the risk of acute kidney injury after contrast-enhanced CT has become extremely low in recent years.^14,15^

Therefore, it is necessary to identify patients who may benefit from additional delayed-phase CCT to evaluate CT-ECV. The myocardial properties of patients who underwent CCT were evaluated to clarify the distribution of CT-ECV and the clinical characteristics of patients with elevated CT-ECV. Age, male sex, hs-cTnT and BNP levels, and cardiac remodelling (e.g. reduced LVEF and LV hypertrophy) were correlated with CT-ECV. ECV_CMR_ is reported to be associated with the hs-cTnT and BNP levels, reduced LVEF, and LV hypertrophy^16–18^; however, to the best of our knowledge, this study is the first to report that CT-ECV is associated with biomarker levels and LV hypertrophy. Extracellular volume is a quantitative index that reflects the spread of the extracellular volume and increases with myocardial injury and fibrosis. The addition of CT-ECV to CCT is beneficial in patients with increased cardiac biomarker levels and cardiac remodelling. Therefore, the cause of increased CT-ECV in these patients must be evaluated.

### Usefulness of cardiac amyloidosis diagnosis by extracellular volume by computed tomography

Extracellular volume imaging is used to diagnose certain diseases, track myocardial remodelling, and predict outcomes.^19^ Myocardial infiltration by amyloid fibrils causes extracellular expansion, and cardiac amyloidosis contributes to the diagnosis and prognosis by evaluating ECV.^20^ Transthyretin cardiac amyloidosis is observed in 13% of elderly patients with heart failure with preserved ejection fraction^21^ and in one in seven elderly patients aged ≥75 years with severe aortic stenosis undergoing TAVI.^22^ Recent investigations of the clinical characteristics of wild-type ATTR-CA revealed that the median age at diagnosis was ≥70 years.^23–25^ Wild-type ATTR-CA is more likely to occur in men than in women across all age categories, and the mean age at diagnosis in women is higher than that in men^26^; therefore, men and women aged ≥60 and ≥70 years, respectively, were included in this study.

Extracellular volume ≥ 40% on cardiac MRI is strongly suggestive of cardiac amyloidosis.^7^ However, the optimal cut-off value for diagnosing cardiac amyloidosis via CT-ECV has not yet been established. Extracellular volume by computed tomography cut-off values of 33.7% and 37% are useful for diagnosing cardiac amyloidosis in patients with severe aortic valve stenosis and suspected heart failure or cardiomyopathy, respectively.^27,28^ Therefore, the cut-off value of abnormal ECV was set at 35% in this study. If the CT-ECV cut-off value is set as 40% in this study, the prevalence of ATTR-CA in patients with CT-ECV ≥ 40% rises to 31% (11/36); however, four of the 15 patients with ATTR-CA who had CT-ECV ≤ 40% were overlooked. Thus, considering the findings of the present study, an ECV ≥ 35% may warrant further testing as a diagnostic threshold. Extracellular volume ≥ 35%, combined with red flags (e.g. carpal tunnel syndrome and increased hs-cTnT) of ATTR-CA, will lead to early diagnosis of ATTR-CA in patients undergoing CCT for coronary artery evaluation.

### Incidental diagnosis of cardiac amyloidosis by cardiac computed tomography

Approximately 1% of patients who undergo bone scintigraphy for non-cardiac evaluation are incidentally diagnosed with ATTR-CA.^29,30^ In contrast, ATTR-CA is incidentally diagnosed in 15% of patients undergoing pre-TAVI planning CCT.^4^ The prevalence of incidental diagnosis of cardiac amyloidosis was 1.7% in this study, which is similar to the results of incidental diagnosis using bone scintigraphy. Transthyretin cardiac amyloidosis is potentially present in elderly patients with heart failure, and the incidental prevalence of ATTR-CA is inevitably elevated in elderly patients with aortic stenosis indicated to undergo TAVI. Thus, the rate of incidental diagnosis of ATTR-CA using imaging is strongly influenced by the patient background. Cardiac amyloidosis screening should be recommended for patients with a high prevalence of cardiac amyloidosis, such as elderly patients and patients with heart failure and LV hypertrophy who have incidentally elevated CT-ECV. Similarly, high CT-ECV in the pre-atrial fibrillation ablation planning CCT leads to the diagnosis of ATTR-CA.^6^ The assessment of CT-ECV and the prevalence of cardiac amyloidosis with pre-atrial fibrillation ablation planning CCT must be investigated in future studies.

## Study limitations

First, this was a single-centre, retrospective study. Validation with a similar cohort would support these results. However, this is the first cohort study with high CT-ECV in an adequate number of patients who underwent CCT for coronary artery evaluation. Further prospective studies with more patients are required to validate these results. Second, the evaluation of monoclonal proteins and bone scintigraphy was not performed in all cases with high CT-ECV. Therefore, cardiac amyloidosis may have remained undiagnosed. Cardiac magnetic resonance was not performed routinely in patients with increased CT-ECV as a significant correlation has been observed between CT-ECV and MRI-ECV in patients with cardiac amyloidosis.^31^ In contrast, five of the 15 patients with cardiac amyloidosis were diagnosed via echocardiography based on previous reports^12^ and did not undergo the process recommended by the algorithm for diagnosing cardiac amyloidosis. Thus, these patients may not have had amyloidosis. Lastly, delayed-phase images were obtained 7 min after the first contrast injection and were performed during the diastolic phase. The ECV during the diastolic phase is 2–3% lower than that during the systolic phase^32^; however, whether diastolic or systolic phase reconstruction is more appropriate for ECV remains unclear.^33^ The protocol for CT-ECV has not been determined and differs among facilities. Lastly, elderly patients (men and women aged ≥60 and ≥70 years, respectively) were included in the present study as cardiac amyloidosis is observed in elderly patients; thus, data for middle-aged patients with low-to-intermediate coronary artery disease risk were not considered.

## Conclusion

Cardiac computed tomography was useful for the evaluation of coronary arteries and the simultaneous evaluation of myocardial properties by CT-ECV. Elevated CT-ECV is associated with elevated cardiac biomarker levels and structural changes in the myocardium, which aid in the diagnosis of cardiomyopathy. Extracellular volume by computed tomography may lead to an incidental diagnosis of cardiac amyloidosis.

## Data Availability

The data underlying this article will be shared on reasonable request to the corresponding author.

## Lead author biography

Tetsuya Oguni is a 36-year-old doctor working in Kumamoto University, Kumamoto, Japan. He performed a fellowship in Saitama Medical University International Medical Center, Saitama, Japan. His areas of interest include heart failure and cardiac imaging.
